# Factors associated with maternal overall quality of life six months postpartum: a cross sectional study from The Norwegian Mother, Father and Child Cohort Study

**DOI:** 10.1186/s12884-021-04303-5

**Published:** 2022-01-03

**Authors:** Lisbeth Valla, Sølvi Helseth, Milada Cvancarova Småstuen, Nina Misvær, Randi Andenæs

**Affiliations:** Department of Nursing and Health Promotion, Faculty of Health Sciences, Metropolitan University, Oslo, Norway

**Keywords:** Life quality, Postpartum period, The Norwegian Mother, Father and Child Cohort Study

## Abstract

**Background:**

Having good Quality of Life (QoL) is essential, particularly for women after childbirth. However, little is known about the factors associated with maternal QoL after giving birth. We aimed to investigate the relationship between characteristics of the mother (socio-demographic variables), selected symptoms (depression and joy/anger), health perception (perception of birth) and possible characteristics of the environment (infant temperament, colic, sleep, parental relationship), with mothers’ overall quality of life when the child is 6 months of age.

**Methods:**

This study is based on the Norwegian Mother, Father and Child Cohort Study (MoBa), conducted at the Norwegian Institute of Public Health from June 1999 to December 2008, which included a total of 86,724 children. Maternal QoL was assessed by the Satisfaction With Life Scale. Joy and anger were measured using the Differential Emotional Scale, mothers’ mental health was assessed using the Edinburgh Postnatal Depression Scale and satisfaction with relationship was measured using the Relationship Satisfaction Scale. Child temperament was measured using the Infant Characteristics Questionnaire and colic, sleep duration and feelings related to childbirth were assessed by mothers’ reports. The associations between life satisfaction and selected variables were analysed using stepwise multiple linear regression models, and the results are presented as effect sizes (ES).

**Results:**

Maternal feelings of joy of having a baby (ES = 0.35), high relationship satisfaction (ES = 0.32), as well as having a baby with normal sleep (ES = 0.31), are factors associated with higher maternal overall QoL. Postnatal depression was negatively associated with mothers’ QoL, and infant colic or child’s temperament (fussiness) showed no such association with mothers’ QoL.

**Conclusions:**

Health professionals and clinicians should focus on infants sleep but also on supporting joy of motherhood and strengthening relationships of the new parents when they develop health interventions or provide counselling to new mothers and their families.

## Background

Giving birth and caring for a new infant mark an important transition in life. Although this transition is often exciting and rewarding, it is also a time of vulnerability and many challenges including sleep disruption [1] and heightened psychosocial stress [2], which in turn pose a risk to successful emotional and psychological adjustments that ensure quality of life [3]. Theorists have conceptualised quality of life (QoL) in multiple ways, including satisfaction with life [4], subjective well-being [5], and happiness [6]. In medical and health science literature, the term ‘health-related quality of life’ is most commonly used [7]. Wilson and Cleary propose a theoretical framework for QoL and suggest that symptoms, functional status, health perception, personal factors and environmental characteristics, in addition to biological and physiological factors, are related to an individual’s overall quality of life. In the present study, we use the term QoL throughout this article to characterise a broad representation, and define overall quality of life in accordance with Diener (1984) as subjective well-being, relating to an evaluation of how people feel and think about their lives [8, 9]. QoL constitutes an important goal for individuals and societies with positive consequences for population health [10, 11]. However, the overall QoL of mothers may be of particular significance, as it may not only affect the mother herself but also the way she interacts with her child and the environment [12].

Several factors impact maternal QoL postpartum. Issues mentioned in the literature such as infant temperament or fussiness, are likely to affect maternal well-being [13]. Infant colic and poor sleep behaviour can give rise to negative emotions in the mother that overshadow the joy of being a mother. Colic is infant fussing and crying that occurs in the infant’s second or third week of life, lasts more than 3 h a day and gradually resolves in the third or fourth month [14, 15]. A study of mothers of new-born’s (*N* = 78) reported that those with an infant with colic had more symptoms of depression and lower well-being [16]. During the postpartum period, most women experience significant disturbances in their sleep patterns due to hormonal changes and responsibility for the care of the new-born [17]. Postpartum sleep quality is a critical health index and sleep deprivation is the main source of stress, anxiety, and depression for postpartum women [18, 19]. In addition, women’s QoL after childbirth may also be affected by factors related to pregnancy and delivery [20–22] and their marital relationship [3, 23, 24]. Research indicates that women who perceived they were ‘very well’ cared for during delivery, and women who felt their overall birth experience was ‘very positive’, were significantly more likely to experience high postnatal functioning [21]. On the other hand, women with a negative childbirth experience are at an increased risk of psychological maladjustment in the first 5 months after giving birth [22].

The majority of previous research focusing on health and quality of life are about the immediate postnatal [21] or postpartum period [25, 26], or risk populations, such as premature birth [27].

There is a paucity of knowledge regarding the role of individual or environmental factors in relation to mothers’ QoL after the puerperal period, from large, unselected populations. To provide a rich picture of the factors influencing maternal quality of life, the aim of the present study was to investigate the relationship between characteristics of the mother (i.e. socio-demographic variables), selected symptoms (i.e. depression and joy/anger), health perception (i.e. perception of birth), and possible characteristics of the environment (i.e. infant temperament, colic, sleep, parental relationship), with mothers’ overall quality of life when the child is about 6 months.

## Methods

### Participants and sample

The present study used data from the Norwegian Mother, Father and Child Cohort Study (MoBa), conducted by the Norwegian Institute of Public Health from 1999 through 2008 [28].

All pregnant women in Norway were eligible to participate in MoBa if they were able to read Norwegian. The MoBa cohort included 114,500 children and 95,200 mothers yielding a response rate of around 41% [28]. Written informed consent was obtained from all MoBa participants upon recruitment and participants did not receive any financial compensation. The establishment of MoBa and the initial data collection were based on a licence from the Norwegian Data Protection Agency and approval from the Regional Committees for Medical and Health Research Ethics. The MoBa cohort is now based on regulations related to the Norwegian Health Registry Act. The current study is based on version 10 of the MoBa data files and was approved by the Regional Committees for Medical and Health Research Ethics (REK-SØ no 9869). Written informed consent was obtained from all MoBa participants. The sample in the present study constitutes 86,724 mothers and children. In the present study twins (*n* = 1480) and triplets (n = 14) were excluded.

### Measurements

#### Overall quality of life

Quality of life was measured using the *Satisfaction With Life Scale (SWLS)* [8] 6 months postpartum. The SWLS includes five items, such as ‘The conditions of my life are excellent’ and ‘I am satisfied with my life’, rated on a Likert scale ranging from 1 = Strongly Disagree, to 7 = Strongly Agree. Mean scores of the five items were computed. The scale revealed a satisfactory internal consistency, with α value 0.89. Several studies have confirmed the validity of the SWLS [8, 29].

#### Socio-demographic data

Information on child gender, gestation age and maternal age, maternal educational level, marital status, work status and experience of birth was collected from the MoBa study at 16–20 weeks of pregnancy and 6 months postpartum.

### Symptoms

#### The Edinburgh postnatal depression scale (EPDS)

Mothers’ mental health was assessed using the *Edinburgh Postnatal Depression Scale (EPDS)*. The EPDS has a sensitivity of 86% and a specificity of 78% [30]. Items are scored on a Likert scale ranging from 0 (‘no, never’) to 3 (‘yes, almost all the time’). A 6-item version was used in the MoBa study. The maximum score is 18, with high scores indicating higher levels of postnatal depression. The internal consistency: Cronbachs α was 0.84 [28].

#### The differential emotional scale (DES)

*The Differential Emotional Scale* [31] consists of a series of subscales that capture various emotions. In the present study, items were selected from the Enjoyment and Anger subscales (DES-IV), and included 3 items of Joy and 3 items of Anger. The respondents rated how often they felt the different emotions on a scale from 1 to 5 where 1 = seldom/never, and 5 = very often. Mean scores were computed. The DES has been widely used in research on self-rated affect and has shown good psychometric properties [31, 32].

#### Feelings related to childbirth

Feelings related to childbirth were assessed 6 months postpartum based on the single statement *‘I felt safe and in good hands’*, with the following response options; applies well, applies partly, does not apply.

### Environment

#### Child’s sleep

Sleep duration at 6 months was assessed using the question, ‘How many hours does your child sleep per day’. Response categories were 10 h or less, 11 to 12 h, 13 to 14 h, and 15 h or more. We defined that the normal sleep of a child aged around 6 months was 13 h or more a day.

#### Infant colic

Colic was measured at 6 months using one question in the questionnaire, ‘*Has your child had the following illness/health problem? Infant colic?’* The response categories were yes or no. Parents thus answered yes if they thought their child had had colic, and no other diagnostic criteria were needed.

#### Child temperament

Child temperament was measured at 6 months using the *Infant Characteristics Questionnaire (ICQ)* [33]. The original ICQ consists of 24 items describing infant behaviour. Four subscales have been identified; Fussy/Difficult, Unadaptable, Dull and Unpredictable, with varying internal consistency previously reported (.79, .75, .39, .50) (Bates et al. 1979). The scale used in MoBa is the Fussy/Difficult scale (8 items). Mothers rank each item on a 7-point Likert scale [1–7], indicating the level of perceived difficulty of handling the described behaviour. Responses to negatively framed questions on the Fussy/Difficult scale were reversed, so that lower scores reflect more difficulty. The total score ranged from 7 to 49 and the internal consistency was 0.69.

#### Satisfaction with relationship

Satisfaction with relationship was measured using the *Relationship Satisfaction Scale* (RS) [34]. RS is a 10-item scale developed for the MoBa study based on previously developed scales. The scale is validated, and showed good psychometric properties [34]. The RS refers to a ‘partner’ instead of being limited to marital spouses and includes the items ‘I am satisfied with the relationship with my partner’, ‘My partner and I have problems in our relationship’, ‘I am very happy in my relationship’, ‘My partner is generally understanding’, and ‘We agree on how children should be brought up’. Responses were rated on a 6-point Likert scale ranging from 1 = *Strongly Disagree* to 6 = *Strongly Agree*. Negative responses were reversed, and items added to form a sum score with a maximum score of 60.

### Statistical analysis

Data are described with the means and standard deviations (SD) for continuous variables and with frequencies and percentages for categorical data. The associations between life satisfaction and selected variables were analysed using stepwise multiple linear regression models. Variables that were associated with QoL – based on the Wilson & Cleary model and our clinical knowledge – were included in the multiple regression model. The results are presented as standardized regression coefficients, eg effect sizes as the analysed covariates are measured on very different scales. The Effect size are interpreted as follows: ES < 01 is considered small, ES > 0.3 moderate and ES > 0.5. The assumptions for linear regression were fulfilled as the residuals were normally distributed. All analyses were considered exploratory, so no correction for multiple testing was performed and *p*-values < 0.05 were considered statistically significant. All statistical analyses were carried out using SPSS, version 25 (IBM Corp, Armonk, NY).

## Results

### Sample characteristics

The participants’ characteristics are described in Table 1. The mean age of included mother was 29.8 and over two thirds had medium to high education. A great majority (83.1%) were employed and almost all were married or co-habiting (97.9%).

**Table 1:** Characteristics of the study population when the child is six months

Child (***N*** = 86,724) mean (SD)	n (%)	mean (SD)	Median (range)
Male **n (%)**	40,205 (51.0)		
Missing (*n* = 7947)			
Gestation age			
≥37 weeks	70,815 (95.0)		
32–36 weeks	3231 (4.3)		
28–31 weeks	309 (0.4)		
< 28 weeks	192 (0.3)		
Missing (*n* = 12,177)			
Birth weight in grams	78,816		3600 (500, 5960)
Missing (*n* = 7908)			
**Mother (N = 86,724)**			
Maternal age	82,377	29.8 (4.5)	
Missing (*n* = 4387)			
Maternal education ^a^			
low	26,607 (34.0)		
medium	32,941 (42.0)		
high	18,836 (24.0)		
Missing (*n* = 8340)			
Work status ^b^			
Education	4930 (6.0)		
Employed	68,153 (83.1)		
Other	8938 (10.9)		
Missing (*n* = 4703)			
Marital status ^c^			
Married/co-habiting	80,333 (97.9)		
Missing (*n* = 4640)			

^a^Education at 16–20 weeks of pregnancy: low education = completed primary school and or lower and upper secondary school. Medium education = completed college / university 1–3 years. High education = completed college / university 4 years

^b^Work Status at 16–20 weeks of pregnancy: education = student, at home, trainee, military service. Employed = employed in the public or private sector. Other = unemployed

^c^Marital status at 16–20 weeks of pregnancy

The proportions of infant girls and boys were similar, with 51.0% of included babies being boys. Almost all of the included babies were born after 37GA (95%), thus the mean birth weight was 3600 g (Table 2).

**Table 2:** Description of study variables

Variable (scoring)	Scale	Range	Mean	SD
Overall quality of life (high scores = higher QoL)	SWLS	5–35	28.8	4.9
Missing *n* = 1684				
Depression (high scores = more depression)	EPDS	0–18	3.1	3.2
Missing *n* = 1710				
Joy (high scores = more joy)	DES-J	2–15	11.9	2.1
Missing *n* = 9109				
Anger (high scores = more anger)	DES-A	2–15	6.2	2.2
Missing *n* = 9131				
Fussiness (higher scores = more fussy)	ICQ	7–49	16.4	5.6
Missing = 4321				
Relationship satisfaction (higher scores = higher satisfaction)	RS	10–60	52.6	7.2
Missing = 6308				
			**%**	
Feeling safe during birth				
applies well			84.5	
applies partly			13.3	
does not apply			2.2	
Missing *n* = 2333				
Normal sleep of child (13 h or more a day)			92.6	
Missing = 3695				

Results from the stepwise linear regression analyses estimating associations between selected factors and mothers’ overall life satisfaction are presented in Table 3. As described in the method section, each block of covariates was added step by step. In block 1, lower age of the mother, higher level of education and living alone were all associated with higher life satisfaction scores. Age and educational level remained significant throughout the regression analyses. The second step of the linear regression analysis, adjusted for confounding variables such as age, educational level and marital status, showed that low depression scores, higher feelings of joy, lower feelings of anger and higher feelings of having been taken care of during birth, were all significantly related to higher life satisfaction.

**Table 3:** Stepwise multivariate linear regression analysis (standardised beta coefficients) with mothers’ quality of life as the dependent variable

Independent Variables	Effect size	*P*-value
*Block 1*		
Age, years	−0.06	< 0.001
Level of education (1 – 3)	0.13	< 0.001
Marital status (not married/cohabitating as ref.)	− 0.02	< 0.001
*Block 2*		
Age, years	−0.02	< 0.001
Level of education (1 – 3)	0.09	< 0.001
Marital status (not married/cohabitating as ref.)	−0.02	< 0.001
Depression (EPDS 6-item) (high scores = more depression)	−0.15	< 0.001
Joy (DES-J) (high scores = more joy)	0.48	< 0.001
Anger (DES-A) (high scores = more anger)	−0.13	< 0.001
Feeling safe during birth (lower scores = more confidence)	− 0.03	< 0.001
*Block 3*		
Age, years	−0.01	0.12
Level of education (1 – 3)	0.08	< 0.001
Marital status (not married/cohabitating as ref.)	−0.001	0.76
Depression (EPDS 6-item) (high scores = more depr.)	−0.12	< 0.001
Joy (DES-J) (high scores = more joy)	0.35	< 0.001
Anger (DES-A) (high scores = more anger)	−0.06	< 0.001
Feeling safe during birth (lower scores = less confidence)	−0.02	< 0.001
Colic (no colic as ref.)	−0.004	0.18
Child’s temperament (ICQ) (lower scores = less fussy)	−0.006	0.06
Child sleeping more than 13 h a day (< 13 h as ref.)	0.31	< 0.001
Relationship satisfaction (RS) (higher scores = more satisfaction)	0.32	< 0.001

*R*^2^ = 0.49

The third step of the analysis, during which the environmental factors were entered, and the model was adjusted for variables from step 2, showed that neither infant colic nor the child’s.

temperament were no longer significantly associated with mothers’ life satisfaction, while children sleeping 13 h or more a day remained statistically significant. With ES = 0.31 the effect of sufficient infant’s sleep on mother’s QoL was moderate. Living alone lost its significant association, while higher level with partnership satisfaction scores was significantly associated with higher QoL. Women who were happy in their relationship exhibited a moderate effect size of this variable with QoL (ES = 0.32). Moreover, joy of motherhood was also significantly associated with QoL, reaching a moderate effect size (ES = 0.35). Finally, the effect of depression was negative and regarded as small with (ES = 0.12). The final model explained 49% of the variance in mothers’ quality of life.

## Discussion

Our results, from a large Norwegian representative sample 6 months after birth, suggest that feelings of joy of having a baby, high relationship satisfaction, as well as having a baby with normal sleep, are factors associated with higher maternal overall QoL. Postnatal depression was negatively associated with mothers’ QoL, and infant colic or child temperament (fussiness) showed no such association with mothers’ QoL.

In accordance with previous studies, our results indicated that positive emotions such as feelings of joy of motherhood are an important variable in maternal overall QoL [3, 35–37]. One explanation for these findings may be that joy and positive emotions in motherhood serve as a buffer against stress [38, 39], and are important components of connectedness in the parent-child attachment that enhances psychological health and well-being [40]. Wilson and Cleary (1995) theorised that psychological symptoms and emotions influence physical or social functioning, role functioning, mental health and general health perceptions [7]. Thus, the feelings of joy mothers experience as a result of having children are likely to contribute to their global happiness [7, 9]. Consequently, children may benefit as well. Indeed, joy and positive emotions in the parent-child interaction have been associated with positive outcomes for the child [12]. However, our results showed that postnatal depression was negatively associated with mothers’ overall QoL. A majority of studies emphasise the postpartum period as a particularly vulnerable time during which women are at increased risk of mental disorders [41–43], and that postpartum depression has negative consequences on maternal QoL [44].

Maternal QoL was also positively affected by environmental characteristics such as high relationship satisfaction and infant sleep quality (13 h or more a day at 6 months). Previous research has demonstrated that relationship dissatisfaction [3, 22–24, 45], and poor infant sleep quality are associated with higher maternal depression scores [46–48] and lower QoL [3]. Nelson et al. (2014) proposed in a previous review that parents of young children are unhappy to the extent that they encounter sleep disturbance and troubled marriages. Among married/cohabiting couples, the partner is usually the major provider of various types of social support and living in an emotionally supportive relationship may protect their physical and psychological health in demanding situations. A person’s perception of relationship quality may therefore directly affect their wellbeing by eliciting positive emotions or by serving as a buffer against the potentially harmful effects of other external stressors [22].

Notably, neither infant colic nor child temperament (fussiness) showed any association with mothers’ QoL. Although factors such as infant colic and fussiness are highly stressful and strongly associated with maternal depression [49, 50] when they are ongoing, our results show that when the child is 6 months, a history of colic and fussiness has no effect on mothers’ QoL. This may indicate that these problems are transient and that the mother’s QoL at 6 months is not affected by such previous challenges. However, higher levels of maternal social support during pregnancy and postpartum have previously been associated with lower rates of maternal reported infant colic [51].

Our findings have important implications for practice and clinical counselling. First, mothers’ overall QoL is a significant indicator of positive mental health that may affect the parent-child dyad, which in turn may influence the child’s development [10, 12]. Our results indicate that healthcare providers should raise awareness about mothers’ joy of motherhood, children’s sleep habits and encourage parents to look after their relationship. Health professionals should inform both parents of the importance of enhancing partnership relations and partner support. Secondly, our findings highlight the transient nature of the phenomenon of colic, and the short period in which it places a burden on parents. Our findings may be considered in the design of effective health promotion strategies that enable new mothers to maintain optimal health and QoL for themselves and their infants. Identifying health promotion factors that may be particularly positive for life satisfaction in this phase may enable policymakers to prioritise and develop interventions or programmes or provide social support that will benefit individuals and/or families.

Although the present study has many strengths, including a large national sample, the results must be interpreted in light of several methodological limitations. First, the assessment of feelings related to childbirth, sleep or colic did not include validated or objective measures. The results may also be impacted by a selection bias because of attrition in the MoBa study, such as higher maternal age, and fewer health-related risks among those who did not participate in the study [28]. Hence, the results must be interpreted with some caution.

## Conclusions

In conclusion, the present study’s results show that joy of motherhood, relationship satisfaction and infant sleep are the most important factors for mothers’ overall QoL 6 months after birth, while a history of infant colic showed no association. Health professionals and clinicians should focus on these issues when they develop health interventions or provide counselling to new mothers and their families.

## Data Availability

The data that support the findings of this study are available from Norwegian Institute of Public Health, but restrictions apply to the availability of these data, which were used under license for the current study, and so are not publicly available. Data are however available from the author upon reasonable request and with permission of the Norwegian Institute of Public Health.

## Abbreviations

MoBa: The Norwegian Mother, Father and Child Cohort Study
QoL: Quality of Life
